# Genetic and transcriptomic analyses of early-onset colon cancer (EOCC): a *post hoc* analysis of 2973 patients from two adjuvant randomized trials

**DOI:** 10.1016/j.esmogo.2024.100106

**Published:** 2024-11-07

**Authors:** A. Gandini, C. Gallois, H. Blons, C. Mulot, N. Agueeff, C. Lepage, R. Guimbaud, L. Mineur, J. Desramé, B. Chibaudel, A. de Reyniès, T. André, P. Laurent-Puig, J. Taieb

**Affiliations:** 1 Centre de Recherche des Cordeliers, Sorbonne Université, Inserm, Université Paris Cité, Personalized Medicine, Phamacogenomics and Therapeutic Optimization, Paris; 2 Institut du Cancer Paris CARPEM, AP-HP Centre, Department of Gastroenterology and Digestive Oncology, Hôpital Européen Georges Pompidou, Paris, France; 3 Medical Oncology Unit 1, IRCCS Ospedale Policlinico San Martino, Genoa, Italy; 4 Assistance Publique-Hôpitaux de Paris, Department of Biochemistry, Pharmacogenetics and Molecular Oncology, European Georges Pompidou Hospital, Paris Cancer Institute CARPEM, Paris; 5 Department of Genetics and Molecular Medicine, Georges Pompidou European Hospital, APHP Centre, Paris; 6 Hepatogastroenterology and Digestive Oncology Department, Dijon Bourgogne Hospital, University of Burgundy and Franche Comté, Dijon; 7 Oncologie Médicale Digestive Gynecologique, CHU Toulouse, Toulouse; 8 Gastrointestinal and Liver Oncology Unit, St Catherine Institute of Cancer Avignon-Provence, Avignon; 9 Department of Oncology, Jean Mermoz Private Hospital, Lyon; 10 Department of Oncology, Franco-Britannique Hospital, Levallois; 11 Laboratoire SeqOIA, Paris; 12 Sorbonne Université and Department of Medical Oncology, Hôpital Saint Antoine, Paris; 13 Institut du Cancer Paris CARPEM, APHP. Centre, Department of Biology, Hôpital Européen Georges Pompidou, Paris, France

**Keywords:** colorectal cancer, early-onset colorectal cancer, genetic profile, transcriptomic profile, CMS

## Abstract

**Background:**

Despite decreasing colon cancer (CC) incidence worldwide, an inverse trend is being registered since the late 1990s in people <50 years old, possibly due to an increasing exposure to lifestyle-related risk factors/exposome. Early-onset CC (EOCC) typically manifests in more advanced stages and may harbor a distinct genetic profile. However, its prognosis remains controversial, and disease stratification through gene expression-based subtyping could potentially provide insight.

**Materials and methods:**

We collected data from stage III CC patients enrolled in PETACC-8 and IDEA-France and we analyzed them according to a cut-off of 50 years of age, defining EOCC and late-onset CC (LOCC). Molecular analyses were carried out to evaluate mismatch repair deficiency (dMMR) and extended genetic profile. RNA-sequencing analyses were carried out to determine consensus molecular subtypes (CMS).

**Results:**

A total of 2607 LOCC and 366 EOCC were included. When compared to LOCC, EOCC were in better general conditions and presented more advanced-stage diseases. EOCC were more often dMMR, CMS1, *RAS* wild-type, and mutated for *PTEN, CTNNB1, ERBB2,* and *DDR2*. Relapse-free survival (RFS) was similar in the two age groups (3-year rate 74% versus 76%), but differences were observed according to CMS. In CMS1, a better RFS was observed in mismatch repair-proficient (pMMR) EOCC versus LOCC (3-year rate 82% versus 63%, *P* = 0.041), while this was not observed in dMMR CMS1.

**Conclusions:**

In conclusion, EOCCs are diagnosed in more advanced stages, are more often dMMR, harbor a specific genetic profile, and have similar RFS when compared to LOCC, even if the age effect on RFS appeared to vary among CMS groups.

## Highlights

• Compared to LOCC, EOCC had better ECOG PS and more advanced-stage diseases.
• EOCC was more often dMMR, CMS1, and *RAS* wild-type.
• EOCC harbors a higher rate of *PTEN, CTNNB1, ERBB2,* and *DDR2* mutations.
• RFS was similar between EOCC and LOCC (3-year rate 74% versus 76%).
• In CMS1, a better RFS was observed in pMMR EOCC versus LOCC (3-year rate 82% versus 63%).

## Introduction

Colorectal cancer (CRC) is the third most common cancer in men and the second in women. Even though CRC incidence is decreasing worldwide due to screening programs, in patients <50 years old, defined as early-onset CRC (EOCRC), there is an alarming increase in its incidence, and CRC is now the first cause of cancer-related death in the United States in males aged <50 years.^1^ The underlying causes are still under investigations, but it appears to be partly related to an elevated exposure to sporadic risk factors, such as obesity, a diet rich in red and processed meat, smoke, and dysbiosis.^2^, ^3^, ^4^, ^5^ EOCRCs are predominantly left-sided^6^ and often present at more advanced stages.^7^^,^^8^ In terms of genetic profile, EOCRCs are more frequently mismatch repair-deficient (dMMR), *RAS/BRAF* wild-type,^9^ and mutated in genes associated with cancer-predisposing syndromes,^10^ even though literature data are not always concordant.^11^ At present, the international consensus on the management of EOCRC (Delphi Initiative for EOCRC—DIRECt Guidelines) recommends applying the same treatment indications as for patients >50 years old both in adjuvant and advanced settings, since a survival benefit with any treatment intensification has not been demonstrated.^12^ However, whether EOCRC represents a distinct biological entity is still under investigation and may have significant clinical implications. By consolidating various gene expression-based classification approaches for CRC, the CRC Subtyping Consortium successfully classified over 4000 CRC samples into four distinct consensus molecular subtypes (CMS) that now serve as a reference for CRC genomic classification.^13^ These subtypes are designated as CMS1, CMS2, CMS3, and CMS4, with common clinical, molecular, and prognostic characteristics, but their prevalence and prognostic impact among EOCRC patients are not known. Therefore, more data are needed to better understand this subset of patients. Results of large cohort extended analyses (clinical, genetic, and transcriptomic) would expand knowledge on young CC patients, who are always more often present in daily practice. Treating young patients is very hard for all the oncologists also from a psychological point of view, and a lot of social and family aspects need to be considered. Collective effort is key to obtaining results that would increase survival and quality of life of young patients. In order to better understand this subgroup of patients, we studied localized early-onset colon cancer (EOCC) from two multicenter randomized trials from a clinical, genetic, and transcriptomic perspective. Analyses were conducted on patients enrolled in PETACC-8^14^ and IDEA-France,^15^ two randomized phase III trials on adjuvant therapy in stage III colon cancer (CC).

The objective of this work was to identify possible differences between stage III EOCC and late-onset CC (LOCC) in terms of clinical, genetic, and transcriptomic characteristics, and evaluate their impact on disease prognosis.

## Materials and methods

### Study population

We included all patients with biological informed consent signed and available tumor blocks of resected stage III colon adenocarcinoma who have participated in the PETACC-8^14^ and IDEA-France^15^ phase III randomized trials. In PETACC-8, 2550 patients 18-75 years old were enrolled between December 2005 and November 2009 from 340 different centers across Europe. Patients were randomized to receive 6 months of FOLFOX4 (bolus and infusional fluorouracil, leucovorin, and oxaliplatin), or FOLFOX4 plus cetuximab. In June 2008, it was amended to restrict random assignment to patients whose tumors expressed wild-type *KRAS*. In IDEA-France, 2010 patients >18 years old were enrolled between May 2009 and May 2014, from 129 French centers. Patients were randomly assigned to receive 3 or 6 months of chemotherapy with modified FOLFOX6 (infusional fluorouracil, leucovorin, and oxaliplatin) or capecitabine plus oxaliplatin (CAPOX).

The study was conducted according to the Declaration of Helsinki, Good Clinical Practice, and local ethical guidelines. The datasets generated during and/or analyzed during this study are available from the corresponding author upon reasonable request.

### Genetic and transcriptomic analysis

Microsatellite instability, KRAS, NRAS, and BRAF status were previously reported in the PETACC-8 trial,^16^ and the assessment methods were the same in the IDEA-France trial. An extensive genomic profile was performed with next-generation sequencing with a colon lung panel from Thermofischer (Waltham, MA), at the “Centre de Recherche des Cordeliers” in Paris, France.

RNA-sequencing analysis were conducted in the same research center. Tumor RNA was extracted from macrodissected formalin-fixed, paraffin-embedded (FFPE) tissue sections using the QIAsymphony™ RNA kit (Qiagen, Hilden, Germany) in the PETACC-8 trial, and from macrodissected FFPE punch biopsy using the RNeasy™ FFPE kit (Qiagen) in the IDEA-France trial. The PolyA-RNAseq library preparation protocols were carried out using 400 ng of template RNA and the QuantSeq 3’mRNA-Seq Kit FWD for Illumina (Lexogen™, Greenland, NH) according to the manufacturer’s instructions. Libraries were sequenced on NovaSeq6000 (Illumina, San Diego, CA). Bioinformatic analyses were carried out using R version 4.2. FASTQ RNA-seq files were mapped using STAR aligner 2.7.9a and raw read counts were obtained using the Rsubread R package. Before mapping, the genome index was built on GRCh38.p13 human genome. Only samples with >10 000 genes detected were kept for further analyses. Batch correction was carried out using Combat_seq function from the sva R package. Mitochondrial and housekeeping genes were removed from the raw count matrix. Then, for each sample, we scaled the corresponding vector of raw counts: counts were divided by the total number of counts of the vector and multiplied by the median of total number of counts across samples. Then, the scaled data were log2 transformed. Two-sided *P* values <0.05 were considered statistically significant.

### Statistical analysis

Biomarker status was analyzed by investigators blinded to patient outcomes and then transmitted for survival analyses to the data center. Relapse-free survival (RFS) was defined according to DATECAN definition as the time from date of randomization to local and/or metastatic relapse, and/or death due to CC, whichever occurs first.^17^ For comparisons of baseline characteristics, categorical outcomes were analyzed with *χ*^2^ tests and continuous outcomes, with standard parametric or nonparametric tests. Clinical and molecular characteristics of the patients were analyzed according to two age groups: patients <50 years old at the time of the diagnosis were classified as EOCC, while patients >50 years old as LOCC. RFS curves were estimated with the Kaplan–Meier method, and adjustment for risk group was done for calculating the hazard ratio (HR). RFS between EOCC and LOCC was investigated in each CMS group using univariate and multivariate Cox models, by adjusting for risk group (low risk defined as pT1-3 and N1; high risk defined as pT4 and/or pN2), using the survival R package. Interaction tests between age and MMR status for RFS was conducted in each CMS group. All statistical analyses were carried out using the R Studio statistical software package and were not adjusted for multiple comparisons.

## Results

### Clinical characteristics

**Figure 1:**
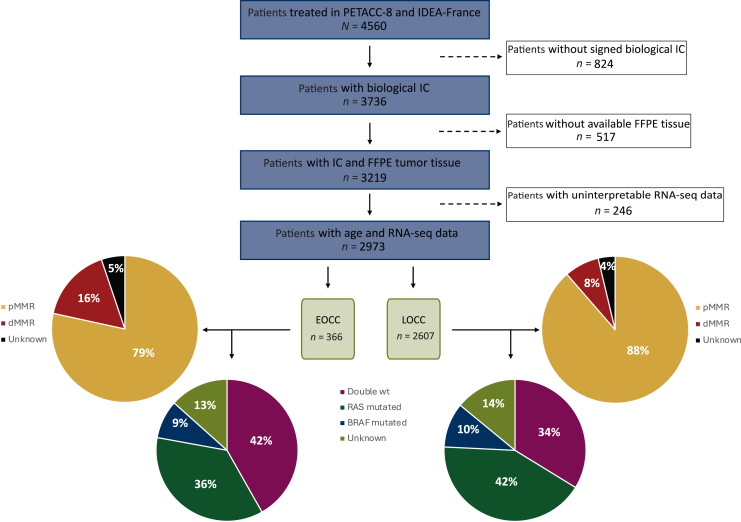
**Flowchart of the study.** dMMR, mismatch repair deficiency; EOCC, early-onset colon cancer; FFPE, formalin-fixed, paraffin-embedded; IC, informed consent; LOCC, late-onset colon cancer; pMMR, mismatch repair proficiency; wt, wild-type.

Among the 4560 patients, clinical and molecular data of 2973 patients (65.2%) were available according to the flowchart shown in Figure 1, including 366 patients (12.3%) with EOCC and 2607 with LOCC (87.7%). Reasons for unavailability of data are described in the flowchart. EOCC patients had more frequently an Eastern Cooperative Oncology Group performance status of 0 (85% versus 78%), bowel obstruction or perforation (24% versus 18%), poorly differentiated tumors (21% versus 13%), and an N2 tumor stage (40% versus 32%) (Table 1).

**Table 1:** Patients’ characteristics in PETACC-8, IDEA-France, and in the pooled analysis of both

	PETACC-8			IDEA-France			Total		
	EO *n* = 275^a^	LO *n* = 1458^a^	*P* value^b^	EO *n* = 91	LO *n* = 1149	*P* value^c^	EO *n* = 366	LO *n* = 2607	*P* value^c^
Sex, *n* (%)			0.3			0.2			0.10
Female	127 (46)	619 (42)		45 (49)	487 (42)		172 (47)	1106 (42)	
Male	148 (54)	839 (58)		46 (51)	662 (58)		194 (53)	1501 (58)	
BMI	24.3	25.5	**<0.001**						
NA	3	13							
Obstr/perf, *n* (%)			0.2			**0.003**			**0.008**
No	214 (78)	1185 (81)		63 (69)	940 (82)		277 (76)	2125 (82)	
Yes	61 (22)	273 (19)		28 (31)	208 (18)		89 (24)	481 (18)	
NA				0	1		0	1	
PS, *n* (%)			**0.014**			0.4			**0.003**
0	233 (87)	1131 (81)		71 (78)	853 (74)		304 (85)	1984 (78)	
1-2	35 (13)	272 (19)		20 (22)	296 (26)		55 (15)	568 (22)	
Unknown	7	55					7	55	
Sidedness, *n* (%)			0.2			0.3			0.5
distal	175 (64)	863 (60)		48 (55)	670 (60)		223 (61)	1533 (60)	
proximal	100 (36)	587 (40)		40 (45)	444 (40)		140 (39)	1031 (40)	
NA	0	8		3	35		3	43	
pT, *n* (%)			0.078			0.7			0.068
pT1-3	205 (75)	1160 (80)		71 (78)	919 (80)		276 (76)	2079 (80)	
pT4	69 (25)	298 (20)		20 (22)	230 (20)		89 (24)	528 (20)	
NA	1	0					1	0	
pN, *n* (%)			**0.035**			0.3			**0.002**
pN1	157 (57)	930 (64)		62 (68)	844 (73)		219 (60)	1774 (68)	
pN2	118 (43)	528 (36)		29 (32)	305 (27)		147 (40)	833 (32)	
Risk group, *n* (%)			**0.002**			0.6			**<0.001**
Low	117 (43)	774 (53)		52 (57)	690 (60)		169 (46)	1464 (56)	
High	157 (57)	684 (47)		39 (43)	459 (40)		196 (54)	1143 (44)	
NA	1	0					1	0	
Grade, *n* (%)			**0.017**			0.3			**<0.001**
G1-2	205 (76)	1185 (82)		76 (89)	1029 (93)		281 (79)	2214 (87)	
G3-4	65 (24)	258 (18)		9 (11)	81 (7.3)		74 (21)	339 (13)	
NA	5	15		6	39		11	54	
MMR status, *n* (%)			**<0.001**			**0.001**			**<0.001**
dMMR	46 (17)	125 (8.7)		14 (17)	75 (7.0)		60 (17)	200 (8.0)	
pMMR	217 (83)	1311 (91)		70 (83)	999 (93)		287 (83)	2310 (92)	
NA	12	22		7	75		19	97	

Low-risk group = pT1-3 and pN1; high-risk group = pT4 and/or pN2. Statistically significant *P*-values are highlighted in bold.

BMI, body mass index; dMMR, mismatch repair deficiency; EO, early onset; IQR, interquartile range; LO, late onset; NA, not available; Obstr, bowel obstruction; Perf, bowel perforation; pMMR, mismatch repair proficiency; PS, performance status.

a Median (IQR).

b Pearson’s chi-square test; Wilcoxon rank sum test.

c Pearson’s chi-square test.

dMMR was significantly more common in EOCC than LOCC (17% versus 8%), and this difference was consistent among the two trials. In the whole population, 260 patients had dMMR tumor, and among them 60 (23%) were <50 years old. dMMR EOCC patients were more likely to be male, while other characteristics were quite balanced among the two age groups as shown in Supplementary Table S1, available at https://doi.org/10.1016/j.esmogo.2024.100106.

### Genetic and molecular classification characteristics

Table 2 shows the extensive genetic profile of EOCC and LOCC included in the trials, according to MMR status. As compared with LOCC, EOCC appeared to be more frequently *KRAS* wild-type (61% versus 55%; *P* = 0.04) and mutated in *PTEN* (8.5% versus 3.8%), *CTNNB1* (6.3% versus 2.5%), *ERBB2* (3.2% versus 1%), and *DDR2* (3.1% versus 0.6%). These differences were mainly due to mismatch repair-proficient (pMMR) EOCC, except for *ERRB2* that was more frequently mutated in both pMMR and dMMR EOCC patients.

**Table 2:** Extensive genetic profiling of patients in PETACC-8, IDEA-France, and in the pooled analysis of both

	All population			pMMR			dMMR		
	EO *n* = 366	LO *n* = 2607	*P* value^a^	EO *n* = 287	LO *n* = 2310	*P* value^a^	EO *n* = 60	LO *n* = 200	*P* value^a^
*TP53*, *n* (%)			0.6			0.7			0.5
M	193 (61)	1402 (62)		171 (67)	1314 (66)		18 (34)	53 (29)	
NM	125 (39)	846 (38)		84 (33)	687 (34)		35 (66)	130 (71)	
NA	48	359		32	309		7	17	
*KRAS*, *n* (%)			**0.044**			**0.022**			**0.002**
M	123 (39)	1006 (45)		101 (40)	946 (47)		21 (40)	35 (19)	
NM	194 (61)	1240 (55)		153 (60)	1052 (53)		32 (60)	148 (81)	
NA	49	361		33	312		7	17	
*NRAS*, *n* (%)			0.3			0.4			0.5
M	9 (2.8)	89 (4.0)		8 (3.1)	83 (4.2)		1 (1.9)	2 (1.1)	
NM	308 (97)	2152 (96)		246 (97)	1911 (96)		52 (98)	181 (99)	
NA	49	366		33	316		7	17	
*BRAF*, *n* (%)			0.3			0.3			**<0.001**
M	32 (10)	266 (12)		26 (10)	167 (8.4)		6 (11)	91 (50)	
NM	286 (90)	1975 (88)		229 (90)	1827 (92)		47 (89)	92 (50)	
NA	48	366		32	316		7	17	
*PIK3CA*, *n* (%)			0.2			0.3			>0.9
M	63 (20)	381 (17)		48 (19)	329 (16)		13 (25)	44 (24)	
NM	254 (80)	1862 (83)		206 (81)	1667 (84)		40 (75)	139 (76)	
NA	49	364		33	314		7	17	
*FBXW7*, *n* (%)			0.3			0.8			0.3
M	31 (9.8)	182 (8.1)		20 (7.9)	150 (7.5)		11 (21)	27 (15)	
NM	286 (90)	2059 (92)		234 (92)	1844 (92)		42 (79)	156 (85)	
NA	49	366		33	316		7	17	
*SMAD4*, *n* (%)			0.5			>0.9			0.3
M	31 (9.8)	250 (11)		28 (11)	223 (11)		2 (3.8)	18 (9.8)	
NM	286 (90)	1993 (89)		226 (89)	1773 (89)		51 (96)	165 (90)	
NA	49	364		33	314		7	17	
*PTEN*, *n* (%)			**<0.001**			**0.003**			0.3
M	27 (8.5)	85 (3.8)		14 (5.5)	46 (2.3)		12 (23)	31 (17)	
NM	290 (91)	2156 (96)		240 (94)	1948 (98)		41 (77)	152 (83)	
NA	49	366		33	316		7	17	
*MAP2K1*, *n* (%)			0.062			**0.032**			>0.9
M	8 (2.5)	26 (1.2)		7 (2.7)	21 (1.1)		1 (1.9)	3 (1.6)	
NM	310 (97)	2218 (99)		248 (97)	1976 (99)		52 (98)	180 (98)	
NA	48	363		32	313		7	17	
*CTNNB1*, *n* (%)			**<0.001**			0.067			0.3
M	20 (6.3)	56 (2.5)		8 (3.1)	30 (1.5)		9 (17)	21 (11)	
NM	297 (94)	2186 (98)		246 (97)	1964 (98)		44 (83)	162 (89)	
NA	49	365		33	316		7	17	
*STK11*, *n* (%)			0.7			0.6			>0.9
M	2 (0.6)	13 (0.6)		1 (0.4)	6 (0.3)		1 (1.9)	6 (3.3)	
NM	315 (99)	2228 (99)		253 (100)	1988 (100)		52 (98)	177 (97)	
NA	49	366		33	316		7	17	
*AKT1*, *n* (%)			0.6			0.5			>0.9
M	2 (0.6)	28 (1.2)		1 (0.4)	21 (1.1)		1 (1.9)	5 (2.7)	
NM	315 (99)	2213 (99)		253 (100)	1973 (99)		52 (98)	178 (97)	
NA	49	366		33	316		7	17	
*ERBB2*, *n* (%)			**0.005**			0.3			0.12
M	10 (3.2)	23 (1.0)		4 (1.6)	18 (0.9)		4 (7.5)	5 (2.7)	
NM	307 (97)	2218 (99)		250 (98)	1976 (99)		49 (92)	178 (97)	
NA	49	366		33	316		7	17	
*EGFR*, *n* (%)			>0.9			>0.9			>0.9
M	2 (0.6)	16 (0.7)		1 (0.4)	10 (0.5)		1 (1.9)	5 (2.7)	
NM	315 (99)	2225 (99)		253 (100)	1984 (99)		52 (98)	178 (97)	
NA	49	366		33	316		7	17	
*FGFR3*, *n* (%)			0.3			**0.050**			0.6
M	4 (1.3)	14 (0.6)		4 (1.6)	9 (0.5)		0 (0)	5 (2.7)	
NM	314 (99)	2229 (99)		251 (98)	1986 (100)		53 (100)	178 (97)	
NA	48	364		32	315		7	17	
*ERBB4*, *n* (%)			0.3			0.4			0.4
M	4 (1.3)	16 (0.7)		3 (1.2)	14 (0.7)		1 (1.9)	1 (0.5)	
NM	313 (99)	2225 (99)		251 (99)	1980 (99)		52 (98)	182 (99)	
NA	49	366		33	316		7	17	
*DDR2*, *n* (%)			**<0.001**			**<0.001**			0.3
M	10 (3.1)	13 (0.6)		8 (3.1)	10 (0.5)		2 (3.8)	3 (1.6)	
NM	308 (97)	2228 (99)		247 (97)	1984 (99)		51 (96)	180 (98)	
NA	48	366		32	316		7	17	
*ALK*, *n* (%)			0.7			0.15			0.3
M	3 (0.9)	17 (0.8)		3 (1.2)	9 (0.5)		0 (0)	6 (3.3)	
NM	314 (99)	2225 (99)		251 (99)	1986 (100)		53 (100)	177 (97)	
NA	49	365		33	315		7	17	

Statistically significant *P*-values are highlighted in bold.

dMMR, mismatch repair deficiency; EO, early onset; LO, late onset; M, mutated; NA, not available; NM, not mutated; pMMR, mismatch repair proficiency.

a Pearson’s chi-square test; Fisher’s exact test.

*BRAF* V600E mutation rate was similar between EOCC and LOCC in the overall population, but higher in LOCC in the dMMR subgroup (50% versus 11%). Focusing on the *KRAS* gene, mutations were more often localized in codon 12 and 13 in both age groups, but codon 146 mutations were more frequent in EOCC pMMR patients (EOCC 16% versus LOCC 7.4%) and those in codon 61 were less frequent in EOCC pMMR patients (EOCC 0.8% versus LOCC 4.7%). Detailed results of *KRAS* mutations in both groups and depending on MMR status are shown in Supplementary Table S2, available at https://doi.org/10.1016/j.esmogo.2024.100106.

Regarding CMS (Table 3), we have classified tumors in terms of a major CMS and a minor CMS, due to high intratumor heterogeneity (tumors related to only one CMS simply have the minor CMS set to not available). By doing so, all tumors get classified and here we report the major CMS only. CMS1 was significantly more frequent in EOCC (24% versus 15%; *P* < 0.001). Overall, CMS2-3-4 were less frequent in EOCC versus LOCC, mainly due to CMS2 characterizing 31% of EOCC versus 36% of LOCC. CMS3 and 4 were very similar in both groups (20% EOCC, 21% LOCC and 45% EOCC, 27% LOCC, respectively). The majority of dMMR patients in both groups were CMS1 (EOCC 74%, LOCC 71%), followed by CMS2 (19% EOCC, 17% LOCC); CMS3 and CMS4 were rare in the dMMR population as expected (<10%).

**Table 3:** CMS distribution according to age group

CMS	PETACC-8			IDEA-France			Total			Total dMMR			Total pMMR		
	EO, *n* (%) *n* = 275	LO, *n* (%) *n* = 1458	*P* value^a^	EO, *n* (%) *n* = 93	LO, *n* (%) *n* = 1159	*P* value^a^	EO, *n* (%) *n* = 368	LO, *n* (%) *n* = 2617	*P* value^a^	EO, *n* (%) *n* = 60	LO, *n* (%) *n* = 201	*P* value^a^	EO, *n* (%) *n* = 289	LO, *n* (%) *n* = 2317	*P* value^a^
**1**	67 (25)	242 (17)	**0.012**	21 (23)	157 (14)	**0.045**	88 (24)	399 (15)	**<0.001**	43 (74)	143 (72)	0.9	40 (14)	239 (10)	0.2
**2**	88 (33)	547 (38)		22 (24)	397 (35)		110 (30)	944 (36)		1 (1.7)	5 (2.5)		107 (37)	915 (40)	
**3**	50 (19)	312 (22)		21 (23)	244 (21)		71 (20)	556 (21)		11 (19)	34 (17)		55 (19)	508 (22)	
**4**	65 (24)	347 (24)		27 (30)	351 (31)		92 (25)	698 (27)		3 (5.2)	18 (9)		85 (30)	645 (28)	
**NA**	5	10		0	0		5	10		2	0		2	10	

Statistically significant *P*-values are highlighted in bold.

CMS, consensus molecular subtypes; dMMR, mismatch repair deficiency; EO, early onset; LO, late onset; NA, not available; pMMR, mismatch repair proficiency.

a Pearson’s chi-square test; Fisher’s exact test.

### RFS according to age groups

With a median follow-up of 6.6 years, 806 patients (27%) had recurrence, and 652 (22%) died.

**Figure 2:**
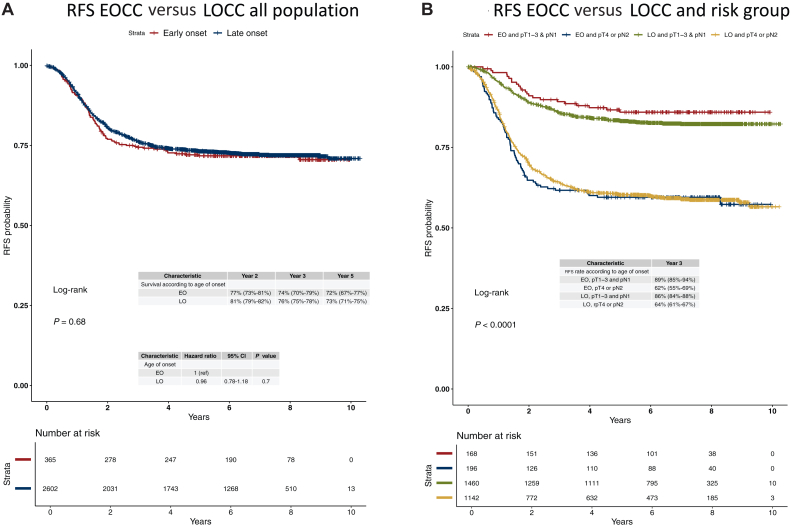
RFS in EOCC versus LOCC of PETACC-8 and IDEA-France trials in (A) all population, and (B) all population according to risk group. CI, confidence interval; EO, early onset; EOCC, early-onset colon cancer; LO, late onset; LOCC, late-onset colon cancer; RFS, relapse-free survival.

RFS was not significantly different in the overall population between EOCC and LOCC (*P* = 0.68) with 3-year RFS of 74% and 76% (Figure 2A), respectively. Similar results were observed when patients were grouped into high and low risk according to pT and pN stage. In high-risk stage III CC patients, 3-year RFS for EOCC and LOCC were 62% versus 64%, as in low-risk patients the 3-year RFS rates were 89% versus 86% (Figure 2B), respectively.

In the multivariable analysis including the main clinical and molecular factors, no association between age group and RFS was observed (HR 1.02; *P* = 0.9), while having a high-risk stage III CC and a *RAS* mutation were associated with shorter RFS (Supplementary Table S3, available at https://doi.org/10.1016/j.esmogo.2024.100106).

**Figure 3:**
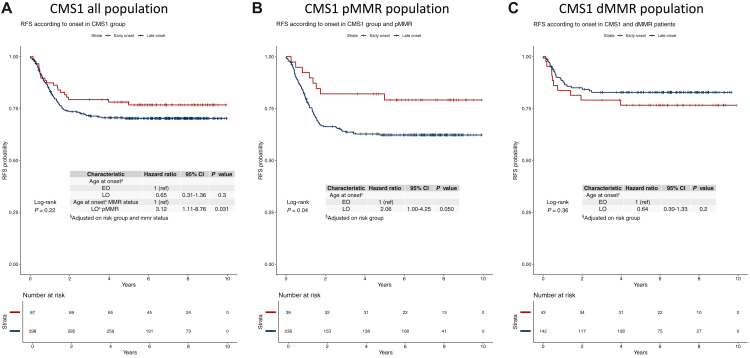
**RFS according to age of onset in the CMS1 group, adjusted for risk group and MMR status.** (A) CMS1 all population; (B) CMS1 pMMR population; (C) CMS1 dMMR population. CI, confidence interval; CMS, consensus molecular subtypes; dMMR, mismatch repair deficiency; EO, early onset; LO, late onset; pMMR, mismatch repair proficiency; RFS, relapse-free survival. ∗Interaction test.

CMS classification was prognostic in our patient population with CMS4 patients having the worse prognosis, and this was true in both EOCC and LOCC (Supplementary Figure S1, available at https://doi.org/10.1016/j.esmogo.2024.100106). In the CMS1 subgroup, after adjustment for risk group and MMR status, there was a non-significant trend for longer RFS in EOCC patients compared to LOCC patients (Figure 3A). The interaction test between age group and MMR status in CMS1 patients was significant, showing a risk of recurrence three times higher in pMMR LOCC. In the subgroup of pMMR and CMS1 patients, EOCC was significantly associated with better RFS [3-year RFS rate 82% in EOCC versus 63% in LOCC (*P* = 0.041)], with a HR adjusted for risk group of 2.06 (*P* = 0.05) (Figure 3B). In the subgroup of dMMR CMS1 patients, an inverse trend was observed, with shorter RFS in EOCC when compared to LOCC (*P* = 0.36), although not statistically significant (3-year RFS rate 79% versus 83%, *P* = 0.36) (Figure 3C). In the other CMS groups, the interaction test of age and MMR status was not significant (Supplementary Figure S2, available at https://doi.org/10.1016/j.esmogo.2024.100106). Additional analyses were carried out to investigate whether age could modify the effect derived from the addition of the anti-epidermal growth factor receptor (EGFR). The curves of the two arms of treatment in PETACC-8 patients according to age and CMS group are available in the supplementary material (Supplementary Figure S3, available at https://doi.org/10.1016/j.esmogo.2024.100106). None of them reach a statistically significant threshold and, moreover, due to the small number of patients included in each subgroup, we cannot reach any solid conclusion.

## Discussion

This is the first comprehensive analysis evaluating and comparing clinical, genetic, and consensus molecular subgroup characteristics of patients with EOCC compared to those with LOCC, from two large-scale phase III adjuvant trials in stage III CC. The percentage of EOCC included was different among the two trials (16% in PETACC-8 and 7% in IDEA-France). This can be explained not only by the upper age limit of 75 years in PETACC-8, but also because an escalation trial (PETACC-8) typically enrolls younger patients than a de-escalation trial (IDEA-France). As expected, EOCC patients were in a better general condition. In our overall study population, we did not observe any difference in the primary tumor location between the two age groups. The literature data rather describe a higher prevalence of left-sided and rectal primary tumors in EOCC, reaching up to 80% of the cases.^11^^,^^18^ The discrepancy may arise from the exclusion of rectal cancers, which are particularly rapidly increasing among young individuals,^19^ as both trials were dedicated to adjuvant treatment of colon cancer only. Similarly, in the sub-analysis of EOCC from the ACCENT database, no differences were found in tumor sidedness.^20^

Multiple studies have reported that EOCC patients were diagnosed with more advanced disease.^21^^,^^22^ Similarly, in our analysis, we observed that clinical presentation in EOCC was more often associated with bowel obstruction or perforation and more advanced pathological stages, especially due to a higher frequency of pN2 stages. It is not currently known whether this is due solely to delayed diagnosis in younger patients^22^ or whether it reflects a more aggressive underlying biology of the tumor itself.^7^ In a pooled analysis of IDEA trials, Fontana et al. found a poorer prognosis for EOCC compared with LOCC despite better adherence to treatment and higher treatment intensity, which may suggest a more aggressive disease biology.^23^ A deep molecular characterization of EOCC seems therefore important to better characterize these tumors.

Among >35 000 patients, Jin et al. found no difference in survival between EOCC and LOCC if molecular biomarkers (MMR status, *BRAF* and *KRAS* mutational status) were taken into consideration.^20^ Two studies^11^^,^^24^ found similar overall genomic landscapes between pMMR EOCC and LOCC, while differences were seen in the dMMR population. However, most of the patients included in these studies had metastatic disease and no transcriptomic data were available. The interest in transcriptomics is rising since mRNA expression correlates with distinct molecular and clinical features^25^ that could possibly drive prognosis and eventually treatment.^26^ To our knowledge, our study represents the largest evaluation of genetic and transcriptomic characteristics of EOCC patients, providing insights into the potential impact of extensive characterization in patient stratification.

As expected, in our study population, dMMR was more frequent in EOCC than LOCC (17% versus 8%), and the lower frequency of *BRAF* mutation (11% versus 50%) in the dMMR EOCC population is probably explained by a higher proportion of Lynch syndrome among young patients.^27^ *KRAS/NRAS* mutations occurred in almost half of the pMMR patients and were more frequent in LOCC than EOCC (49% versus 42%). An inverse rate was observed in the dMMR population, in which LOCC was more often *RAS* wild-type probably due to the higher prevalence of *BRAF* mutation and their mutually exclusive relationship.

We therefore decided to better characterize *RAS* mutations due to their high prevalence in CC and their druggable nature.^28^^,^^29^ It is interesting to notice that *KRAS* G12C mutation was more prevalent in EOCC (14% versus 9% in pMMR patients), as previously reported in a retrospective cohort of metastatic CRC.^30^ *ERBB2* was three times more often mutated in EOCC than LOCC, and, since evidences on its targetable nature are rising,^31^ this could allow more treatment options. However, ERBB2 alterations could also suggest a possible poorer prognosis in this population.^32^, ^33^, ^34^ Available data are for sure not conclusive on these points, but they underline the importance of a complete molecular characterization of EOCC, since it could allow specific targeted therapeutic options in higher numbers of patients. EOCC patients were also more often harboring mutations in genes related to hereditary syndromes, such as *PTEN* and *CTNNB1* (β-catenin1), with an overall higher rate of mutations in the dMMR population, as also previously reported.^35^ Moreover, it is possible that in the EOCC population the wider spectrum of mutations could modulate the anti-EGFR sensitivity/resistance, conferring opposite response to anti-EGFR in EOCC CMS1-2 and CMS3-4 (Supplementary Figure S3, available at https://doi.org/10.1016/j.esmogo.2024.100106).

When looking at our transcriptomic results, we found that CMS1 was more frequent in EOCC. As expected, >70% of the dMMR population was CMS1 in both age groups. Among CMS1 patients, EOCC was more dMMR (48%) than LOCC (37.4%). The prevalence of CMS2-3-4 increases with age, in line with the limited data available in the literature.^9^ Since evidences of the role of CMS in driving prognosis are emerging,^26^ we decided to evaluate it according to age group. As the higher prevalence of high-risk patients and dMMR tumors in EOCC could represent a bias in our prognostic evaluation, we adjusted the analyses for these factors, and we carried out interaction tests between age and MMR status in each CMS group. We therefore focused on the CMS1 group, for which we had a significant interaction test, and surprisingly we observed opposite prognosis according to the MMR status. Thus, compared to LOCC, EOCC showed a significantly better prognosis in terms of RFS in the pMMR CMS1 population (Figure 3B). By contrast, although not statistically significant, prognosis in the dMMR CMS1 population showed a shorter RFS in dMMR EOCC (Figure 3C). Such an important impact of age on the risk of recurrence according to the MMR status and CMS has never been reported before. Lastly, some new insights suggest a possible benefit of immunotherapy in CMS1 pMMR/microsatellites stable patients, that, if confirmed, would open the possibility to use immune checkpoint inhibitors in this selected subgroup of ‘cold’ tumors.^36^

The greater limitation of our research is its *post hoc* nature with significant attrition in patient numbers and quality of samples, which may limit the accuracy of the results. Moreover, no data on germline pathogenic variants were collected. Finally, the cut-off of 50 years of age was used based on literature data that identify the patients as EOCC before the screening age. Exploring different age cut-offs would have been interesting; however, due to the small number of patients in each group, it would have compromised statistical power.

In conclusion, our work confirms that compared to LOCC, EOCC are usually diagnosed in more advanced stages, are more often dMMR, and harbor more often alterations in inheritance-related genes. However, a higher prevalence of *KRAS G12C* mutations and ERBB2 alterations in EOCC suggests the importance of genomic characterization to optimize therapeutic strategies in these patients. RNA-seq provided unique data on its role in stratifying patients, since the age effect on RFS appeared to vary among CMS groups. In CMS1 patients, age <50 years may represent a good prognostic factor among the pMMR patients, while the possible poor prognostic role of age <50 years in dMMR CMS1 needs further investigations.
