# Supplementary material for: Genetic and transcriptomic analyses of early-onset colon cancer (EOCC): a post hoc analysis of 2973 patients from two adjuvant randomized trials
Source: ESMO Gastrointest Oncol. 2024 Nov 7;7:100106. doi: 10.1016/j.esmogo.2024.100106 (PMC12836623; doi:10.1016/j.esmogo.2024.100106)
Supplement: Supplemental Material [file mmc1.docx]

**Supplementary**

|  | **PETACC-8 dMMR** | | | **IDEA-FRANCE dMMR** | | | **Total dMMR** | | |
| --- | --- | --- | --- | --- | --- | --- | --- | --- | --- |
|  | **EO**  **N=46^1^** | **LO**  **N=125^1^** | **p-value^2^** | **EO**  **N=14^1^** | **LO**  **N=75^1^** | **p-value^2^** | **EO**  **N=60^1^** | **LO**  **N=200^1^** | **p-value^2^** |
| **Sex** |  |  | **<0.001** |  |  | 0.3 |  |  | **<0.001** |
| Female | 13 (28%) | 73 (58%) |  | 6 (43%) | 43 (57%) |  | 19 (32%) | 116 (58%) |  |
| Male | 33 (72%) | 52 (42%) |  | 8 (57%) | 32 (43%) |  | 41 (68%) | 84 (42%) |  |
| **Obstr/perf** |  |  | 0.9 |  |  | 0.4 |  |  | 0.6 |
| No | 39 (85%) | 107 (86%) |  | 14 (100%) | 65 (87%) |  | 53 (88%) | 172 (86%) |  |
| Yes | 7 (15%) | 18 (14%) |  | 0 (0%) | 10 (13%) |  | 7 (12%) | 28 (14%) |  |
| **PS** |  |  | 0.4 |  |  | 0.7 |  |  | 0.2 |
| PS 0 | 39 (87%) | 96 (81%) |  | 11 (79%) | 53 (71%) |  | 50 (85%) | 149 (77%) |  |
| PS 1-2 | 6 (13%) | 23 (19%) |  | 3 (21%) | 22 (29%) |  | 9 (15%) | 45 (23%) |  |
| Unknown | 1 | 6 |  |  |  |  | 1 | 6 |  |
| **Sidedness** |  |  | 0.072 |  |  | >0.9 |  |  | 0.10 |
| distal | 13 (28%) | 20 (16%) |  | 2 (14%) | 11 (15%) |  | 15 (25%) | 31 (16%) |  |
| proximal | 33 (72%) | 105 (84%) |  | 12 (86%) | 62 (85%) |  | 45 (75%) | 167 (84%) |  |
| NA |  |  |  | 0 | 2 |  | 0 | 2 |  |
| **pT** |  |  | 0.5 |  |  | **0.034** |  |  | 0.6 |
| pT1-3 | 33 (72%) | 96 (77%) |  | 14 (100%) | 54 (72%) |  | 47 (78%) | 150 (75%) |  |
| pT4 | 13 (28%) | 29 (23%) |  | 0 (0%) | 21 (28%) |  | 13 (22%) | 50 (25%) |  |
| **pN** |  |  | 0.8 |  |  | >0.9 |  |  | 0.7 |
| pN1 | 28 (61%) | 79 (63%) |  | 12 (86%) | 60 (80%) |  | 40 (67%) | 139 (70%) |  |
| pN2 | 18 (39%) | 46 (37%) |  | 2 (14%) | 15 (20%) |  | 20 (33%) | 61 (30%) |  |
| **Risk group** |  |  | 0.2 |  |  | **0.045** |  |  | 0.8 |
| Low | 19 (41%) | 65 (52%) |  | 12 (86%) | 43 (57%) |  | 31 (52%) | 108 (54%) |  |
| High | 27 (59%) | 60 (48%) |  | 2 (14%) | 32 (43%) |  | 29 (48%) | 92 (46%) |  |
| **Grade** |  |  | 0.6 |  |  | 0.7 |  |  | 0.7 |
| G1-2 | 27 (61%) | 70 (57%) |  | 10 (83%) | 53 (74%) |  | 37 (66%) | 123 (63%) |  |
| G3-4 | 17 (39%) | 52 (43%) |  | 2 (17%) | 19 (26%) |  | 19 (34%) | 71 (37%) |  |
| Unknown | 2 | 3 |  | 2 | 3 |  | 4 | 6 |  |
| ^1^n (%) | | | | | | | | | |
| ^2^Pearson's Chi-squared test; Fisher's exact test | | | | | | | | | |

Suppl Table 1: Clinical and molecular characteristics of EOCC vs LOCC among the dMMR population. Abbreviations: EO=Early-onset; LO=Late-onset; PS=Performance status; obstr=bowel obstruction; perf=bowel perforation; NA=not available. Low risk group=pT1-3 and pN1; high risk group=pT4 and/or pN2

|  | **All population** | | | **pMMR** | | | **dMMR** | | |
| --- | --- | --- | --- | --- | --- | --- | --- | --- | --- |
| **KRAS codon** | **EO**  **N=366^1^** | **LO**  **N=2,607^1^** | **p-value^2^** | **EO**  **N=287^1^** | **LO**  **N=2,310^1^** | **p-value^2^** | **EO**  **N=60^1^** | **LO**  **N=200^1^** | **p-value^2^** |
|  |  |  | **0.006** |  |  | **0.007** |  |  | >0.9 |
| **Codon 12**    **Mutation**  **G12A**  **G12R**  **G12D**  **G12C**  **G12F**  **G12S**  **G12W**  **G12V** | 75 (61%)  **N=95***  5 (5.3%)  1 (1.1%)  38 (40%)  11 (12%)  0  2 (2.1%)  1 (1.1%)  17 (18%) | 658 (67%)  **N=836***  50 (6%)  10 (1.2%)  285 (34%)  75 (9%)  2 (0.2%)  51 (6.1%)  0  185 (22%) |  | 67 (66%)  **N=79***  5 (6.3%)  1 (1.3%)  30 (38%)  11 (14%)  0  2 (2.5%)  1 (1.3%)  17 (22%) | 629 (68%)  **N=793**  47 (5.9%)  9 (1.1%)  272 (34%)  73 (9.2%)  2 (0.3%)  50 (6.3%)  0  176 (22%) |  | 8 (40%)  **N=15***  0  8 (53%)  0  0 | 13 (41%)  **N=23***  1 (4.3%)  8 (35%)  1 (4.3%)  3 (13%) |  |
| **Codon 13**    **Mutation**  **G13R**  **G13D**  **G13C**  **G13E**  **G13V** | 20 (16%)  **N=95***  0  19 (20%)  0  0  1 (1.1%) | 178 (18%)  **N=836***  1 (0.1%)  167 (20%)  7 (0.8%)  1 (0.1%)  2 (0.2%) |  | 12 (12%)  **N=79***  0  11 (14%)  0  0  1 (1.3%) | 164 (18%)  **N=793***  1 (0.1%)  153 (19%)  7 (0.9%)  1 (0.1%)  2 (0.3%) |  | 7 (35%)  **N=15***  7 (47%) | 10 (31%)  **N=23***  10 (43%) |  |
| **Codon 61** | 1 (0.8%) | 46 (4.7%) |  | 1 (1.0%) | 42 (4.5%) |  | 0 (0%) | 2 (6.2%) |  |
| **Codon 117** | 2 (1.6%) | 13 (1.3%) |  | 1 (1.0%) | 12 (1.3%) |  | 1 (5.0%) | 1 (3.1%) |  |
| **Codon 146** | 20 (16%) | 73 (7.4%) |  | 18 (18%) | 67 (7.2%) |  | 2 (10%) | 4 (12%) |  |
| **Others** | 4 (3.3%) | 17 (1.7%) |  | 2 (2.0%) | 14 (1.5%) |  | 2 (10%) | 2 (6.2%) |  |
| ^1^n (%) | | | | | | | | | |
| ^2^Fisher's exact test  *Percentage of each mutation are referred to the KRAS-mutated population for which data of specific mutation was available | | | | | | | | | |

Suppl Table 2: Distribution of KRAS codon mutations and specific KRAS G12 and G13 mutations in the pooled analysis of patients in PETACC-8 and IDEA-France, and their variation according to the MMR status. Abbreviations: EO=Early-Onset; LO=Late-Onset; pMMR=proficient mismatch repair system; dMMR=deficient mismatch repair system.

|  | **HR^1^** | **95% CI^1^** | **p-value** |
| --- | --- | --- | --- |
| **Age at onset** |  |  |  |
| EO | — | — |  |
| LO | 1.02 | 0.80, 1.29 | 0.9 |
| **Sidedness** |  |  |  |
| proximal | 0.87 | 0.73, 1.03 | 0.11 |
| **Grade** |  |  |  |
| G3-4 | 1.22 | 0.99, 1.51 | 0.065 |
| **Sex** |  |  |  |
| Female | — | — |  |
| Male | 1.15 | 0.98, 1.35 | 0.079 |
| **Bowel obstr/perf** |  |  |  |
| Yes | 1.19 | 0.99, 1.44 | 0.066 |
| **ECOG PS** |  |  |  |
| PS 1-2 | 1.15 | 0.95, 1.37 | 0.15 |
| **Risk Group** |  |  |  |
| pT4 or pN2 | 3.08 | 2.60, 3.65 | **<0.001** |
| **KRAS & NRAS status** |  |  |  |
| M | 1.41 | 1.19, 1.68 | **<0.001** |
| **BRAF status** |  |  |  |
| M | 0.74 | 0.37, 1.48 | 0.4 |
| **MMR status** |  |  |  |
| dMMR | — | — |  |
| pMMR | 1.24 | 0.83, 1.87 | 0.3 |
| **BRAF status * MMR status** |  |  |  |
| M * pMMR | 1.95 | 0.93, 4.09 | 0.079 |
| ^1^HR = Hazard Ratio, CI = Confidence Interval | | | |

Suppl table 3: Adjusted association between age group and RFS (multivariate Cox models). Abbreviations: EO=Early-onset; LO=Late-onset; Obstr=bowel obstruction; Perf=bowel perforation; NM=not mutated; M=mutated.

Suppl Figure 1: RFS according to age of CMS group in A=all population; B=LOCC; C=EOCC.

Suppl Figure 2: RFS according to age of onset in CMS2-3-4 group, adjusted on risk group and MMR status.

Suppl Figure 3: RFS in the PETACC-8 population according to age and treatment (FOLFOX +/- cetuximab), in each CMS group.
